# Blood Lead and Other Metal Biomarkers as Risk Factors for Cardiovascular Disease Mortality: Erratum

**DOI:** 10.1097/01.md.0000479945.79166.b6

**Published:** 2016-01-29

In the article “Blood Lead and Other Metal Biomarkers as Risk Factors for Cardiovascular Disease Mortality”,^1^ which appeared in Volume 95, Issue 1 of *Medicine*, two license types were originally provided. The article should only have had a CC0 license. Also, Dr. Parker should have been listed as the last author in the byline. The article has since been corrected online.
